# Intra-Venous Lidocaine to Relieve Neuropathic Pain: A Systematic Review and Meta-Analysis

**DOI:** 10.3389/fneur.2019.00954

**Published:** 2019-09-18

**Authors:** Bo Zhu, Xiayun Zhou, Qinghe Zhou, Haiyan Wang, Shougen Wang, Kaitao Luo

**Affiliations:** ^1^Department of Anesthesiology, Zhejiang Chinese Medical University Affiliated Jiaxing Hospital of Traditional Chinese Medicine, Jiaxing, China; ^2^Department of Anesthesiology, Second Affiliated Hospital of Jiaxing University, Jiaxing, China; ^3^Department of Acupuncture, Zhejiang Chinese Medical University Affiliated Jiaxing Hospital of Traditional Chinese Medicine, Jiaxing, China

**Keywords:** neuralgia, causalgia, local anesthetics, pain, adverse events

## Abstract

**Background:** The prevalence of neuropathic pain is estimated to be between 7 and 10% in the general population. The efficacy of intravenous (IV) lidocaine has been studied by numerous clinical trials on patients with neuropathic pain. The aim of this systematic review and meta-analysis was to evaluate the efficacy of IV lidocaine compared with a placebo for neuropathic pain and secondly to assess the safety of its administration.

**Methods:** A literature search on PubMed, Scopus, CENTRAL (Cochrane Central Register of Controlled Trials), and Google scholar databases was performed for relevant studies published up to February 2019. Randomized controlled trials (RCTs) evaluating IV lidocaine treatment for pain relief in patients with neuropathic pain were included.

**Results:** 26 articles met the inclusion criteria. Patients with varied etiology of neuropathic pain were among the patient samples of these studies. Fifteen articles were included for quantitative analysis. Lidocaine was superior to a placebo in relieving neuropathic pain in the early post-infusion period [Mean Difference (MD) = −11.9; 95% Confidence interval (CI): −16.8 to −7; *p* < 0.00001]. Multiple infusions of lidocaine over a period of 4 weeks, however, had no significant effect on reliving neuropathic pain (MD = −0.96; 95% CI: −2.02 to 0.11; *p* = 0.08). IV lidocaine was also associated with a significant number of adverse events compared to a placebo [Odds Ratio (OR) = 7.75; 95% CI: 3.18–18.92; *p* < 0.00001].

**Conclusion:** Our study indicates that while IV lidocaine is effective in pain control among patients with neuropathic pain in the immediate post-infusion period, it does not have a long-lasting, persistent effect. IV infusions of the drug are associated with an increased risk of side effects compared to a placebo. However, the risk of serious adverse events is negligible. Further, well-designed RCTs evaluating the effects of various dosages and infusion periods of IV lidocaine are required to provide clear guidelines on its clinical use.

## Introduction

The International Association for the Study of Pain defines neuropathic pain as “pain resulting from damage to the peripheral or central nervous system (1).” Its prevalence has been reported on several countries worldwide, varying from 3.3% in Austria (2) to 6.9% in France (3) and as high as 8% in the UK (4). van Hecke et al. (5), in their systematic review, report the prevalence of neuropathic pain to be between 7 and 10%. Epidemiological surveys show that a large proportion of patients with neuropathic pain do not receive adequate treatment (6). This may be attributable to clinicians' low diagnostic accuracy and by a lack of knowledge of effective drugs and their proper use (7). Neuropathic pain has a significant impact on quality of life and can be responsible for a substantial financial burden on individuals afflicted.

Lidocaine (lignocaine), a widely used local anesthetic, has been used intravenously (IV) as an antiarrhythmic drug. Reports in the 1950s described the effectiveness of IV lidocaine for pain in cancer and post-operative patients (8). Later, in the 1980s, trials suggested that IV lidocaine is also effective for alleviating neuropathic pain (9, 10). Lidocaine is thought to produce analgesia by exerting a blockade of peripheral and central sodium ion gate channels, including those in the spinal dorsal horn. A number of clinical trials have been conducted to date, evaluating the efficacy of IV lidocaine in patients with neuropathic pain (11–14). However, only one systematic review and meta-analysis, which was published in 2005, has evaluated a pooled treatment effect (15). Since 2005, however, a number of new trials on IV lidocaine have been published in the literature (16–19). Therefore, the aim of this systematic review and meta-analysis was to evaluate the efficacy of IV lidocaine compared with a placebo for neuropathic pain and secondly to assess the safety of its administration.

## Materials and Methods

### Search Strategy

PubMed, Scopus, CENTRAL (Cochrane Central Register of Controlled Trials), and Google scholar databases were searched for relevant studies published up to February 2019. The PICOS (Population, Intervention, Comparison, Outcome, and Study design) outline was used for the electronic search. Keywords used for the patient sample population were: Neuralgia [MeSH] OR neuropathic pain [MeSH] OR pain [MeSH] OR causalgia [MeSH]; for intervention were: lidocaine [MeSH] OR intravenous anesthesia [MeSH] OR lignocaine [MeSH] OR local anesthetics [MeSH] OR intravenous lidocaine [Free text]; for comparison were: saline [MeSH] OR placebo effect [MeSH]; for outcomes were: pain [MeSH] OR analog pain scale [MeSH] OR adverse events [MeSH] OR pain relief [Free text] OR pain assessment [MeSH]. Study designs that were searched were randomized clinical trials (RCTs). We also analyzed references of included studies and pertinent reviews on the topic for the identification of additional studies. Guidelines of the PRISMA Statement (Preferred Reporting Items for Systematic Reviews and Meta-analyses) (20) and the Cochrane Handbook for Systematic Reviews of Intervention (21) were followed during the conduct of the review.

### Eligibility Criteria

We included studies conducted on patients (*Population*) with neuropathic pain from any cause that evaluated the use of intravenous lidocaine (*Intervention)* for pain relief compared with a placebo (*Comparison)* and that assessed pain relief and adverse effects (*Outcomes)*. Studies excluded were: non-english language studies, animal studies, retrospective studies, uncontrolled and non-blinded studies and studies comparing lidocaine with an active drug.

### Data Collection and Analysis

Two reviewers examined the studies based on the inclusion criteria. The studies were reviewed firstly on their title and abstracts, followed by a full-text review of potentially relevant articles. Any difference in opinion between the reviewers was resolved by discussion. Two reviewers then extracted the following data from the studies: general information on the trial (authors, year of publication, study type), number of patients, etiology of pain, lidocaine dosage, placebo used, outcome assessment scale, pain scores, and follow-up period of outcome measurements and adverse events. Attempts were made as needed to contact the corresponding authors via email for any missing data.

### Quality Assessment

The Cochrane Collaboration risk of bias assessment tool for RCTs was used for quality assessment of the included trials (22). Studies were rated as low risk, high risk, or unclear risk of bias for: random sequence generation, allocation concealment, blinding of participants and personnel, blinding of outcome assessment, incomplete outcome data, selective reporting, and other biases. Articles were rated on each of the above items as: low risk of bias (score of 2), unclear risk of bias (score of 1), or high risk of bias (score of 0). The overall quality was then categorized as low (score 0–5), medium (score 6–10), or high (score 11–14).

### Statistical Analysis

Data collected from the included studies was entered into Review Manager (RevMan, version 5.3; Nordic Cochrane Center [Cochrane Collaboration], Copenhagen, Denmark; 2014) for the meta-analysis. Pain scores reported as mean and standard deviation (SD) were used. If no SD was reported, we calculated it from Standard Error of the Mean (SEM) and sample size. If complete data on pain outcomes was not available from the article, a 2005 meta-analysis (15) on this subject was referred to for missing data. The total number of adverse events, as described in the included studies, was pooled. No distinction was made on the severity of each adverse event. Considering the variation in the studies, a random-effects model was used to calculate pooled effect size. Odds ratios (OR) were calculated for adverse events. The evaluation of heterogeneity was calculated using the *I*^2^ statistic. We considered *I*^2^ of <40% as unimportant, while that of more than 40% was viewed as moderate to considerable heterogeneity.

## Results

### Search Results

**Figure 1:**
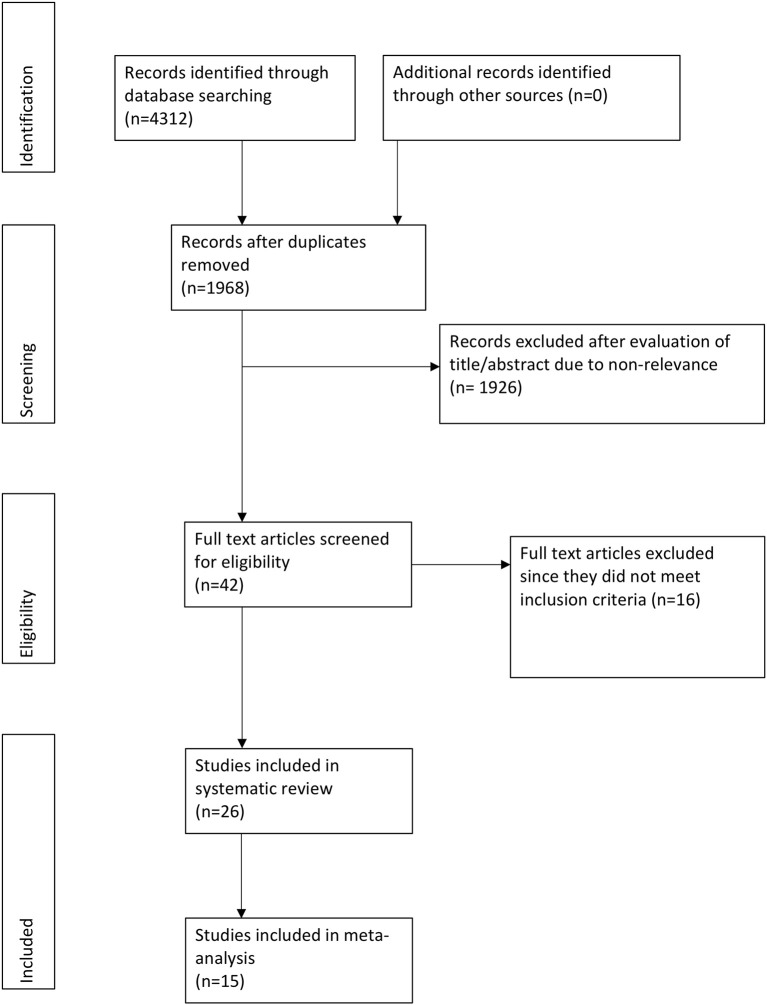
Flowchart of study.

Four thousand three hundred twenty-one records were found after the initial search (Figure 1). Full texts of 42 studies were reviewed, of which 16 studies were excluded: 2 were duplicate publications (23, 24), 4 were non-randomized (25–28), 2 were non-blinded (29, 30), 2 were retrospective studies (31, 32), 2 had used lignocaine for prevention of post-herpetic neuralgia (phn) (33, 34), 3 did not use a placebo (35–37), while the remaining study did not use any controls (38). A total of 26 articles were included in the systematic review (11–14, 16–19, 39–56).

### Quality Assessment

**Figure 2:**
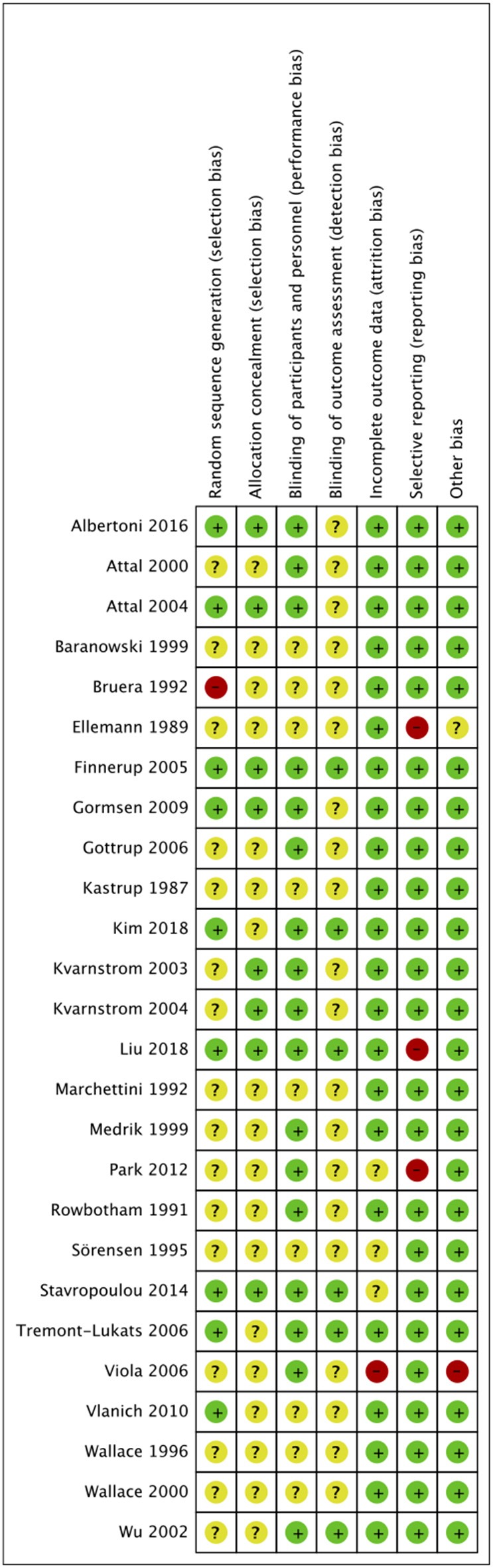
Risk of bias summary.

The risk of bias summary is presented in Figure 2. Methods of randomization were clearly described in 9 studies (16–18, 42, 43, 52–54, 56), allocation concealment was described in eight studies (16, 18, 39, 41–43, 52, 54) and blinding of outcome assessment was clearly mentioned in 6 studies (17, 18, 42, 51, 52, 56). Ten of the studies were categorized as medium quality, while the remaining were rated as high quality trials.

### Characteristics of Included Studies and Data Analysis

Details of the included studies are presented in Table 1. The trial design was parallel in five studies (16, 17, 52, 53, 56), while all the remaining studies were cross-over trials. Patients with various causes of neuropathic pain were studied in the included articles. In one trial of post-amputation pain where phantom and stump pain were studied, only scores of stump pain were included (51). In another trial where spontaneous and evoked pain was studied, we included only the spontaneous pain group (42). The sample size of included studies varied from 10 to 30 participants per group, with the exception of one trial that had a large sample size of 90 patients in the intervention group. However, pain scores in the trial were not reported as mean and SD. The dosage and infusion times of lidocaine also varied across the studies. The duration of lidocaine infusion ranged from 30 min to 6 h. One study (43) used lidocaine injection given over just 1 min. A 10-point or 100-point Visual analog scale (VAS) was used by all studies to assess pain, except for one trial which used the McGill Pain Questionnaire (53). In the majority of studies, pain was assessed in the immediate post-infusion period, i.e., from just after infusion to up to 1–3 days post-infusion. Data from these studies was pooled to evaluate the early effect of lidocaine on neuropathic pain. In three trials (16, 17, 53), lidocaine was infused in 4 sessions over a period of 4 weeks and pain was assessed after the 4th infusion. These three studies were pooled together to evaluate the effect of multiple lidocaine infusions on neuropathic pain.

**Table 1:** Characteristics of included studies.

**References**	**Methodology**	**Cause of pain**	**Evaluable participants**	**Lidocaine dosage**	**Placebo**	**Pain scale used**	**Time of measurement post infusion**	**Pain values as Mean (*****SD*****)**	
								**Lidocaine**	**Placebo**
Kastrup et al. (14)	Crossover 5 weeks washout	Painful diabetic neuropathy	15	Lidocaine 5 mg/kg ×30 min	0.9% saline	100-VAS	1–3 days	27.27 (24.53)	35.4 (29.33)
Ellemann et al. (13)	Crossover 1 week washout	Neuropathic cancer pain	10	Lidocaine 5 mg/kg x 30 min	0.9% saline	10-VAS	Immediately after & at 1 h	NA	NA
Rowbotham et al. (11)	Crossover 2 days washout	PHN	19	Lidocaine 5 mg/kg x 1 h	0.9% saline	100-VAS	Up to 60 min	29.8 (24.5)	43.6 (29.3)
Bruera et al. (12)	Crossover 2 days washout	Neuropathic pain from cancer	10	Lidocaine 5 mg/kg x 30 min	0.9% saline	100-VAS	Immediately after & up to 2 days	36.9 (26)	34.1 (29.8)
Marchettini et al. (45)	Crossover washout not reported	Peripheral neuropathic pain	10	Lidocaine 1.5 mg/kg over 1 min	0.9% saline	100-VAS	At 35 min	59.3 (25)	65 (14)
Sörensen et al. (44)	Crossover 1 week washout	Fibromyalgia	12	Lidocaine 5 mg/kg x 30 min	0.9% saline	100-VAS	Up to 60 min	NA	NA
Wallace et al. (49)	Crossover 1 week washout	Neuropathic pain from peripheral nerve injury	11	Lidocaine IV infusions targeted to deliver plasma concentrations of 0.5, 1.0, 1.5, 2.0, and 2.5 mcg/ml	0.9% saline	100-VAS	Till post infusion	24.82 (19.61)	55.1 (36.66)
Baranowski et al. (50)	Crossover 1 week washout	PHN	24	Lidocaine IV at 1 and 5 mg/kg x 2 h	0.9% saline	100-VAS	Till post infusion	17.5 (31.35)	10.08 (27.24)
Medrik et al. (47)	Crossover 2–7 day washout	Painful lumbosacral radiculopathy	30	Lidocaine 5 mg/kg IV x 1–2 h	0.9% saline	100-VAS	Up to 1 h	31 (27.39)	38 (27.39)
Attal et al. (48)	Crossover 3 weeks washout	Neuropathic pain from stroke and spinal cord injury	16	Lidocaine 5 mg/kg x 30 min	0.9% saline	100-VAS	Till post infusion	31 (28)	46 (24)
Wallace et al. (40)	Crossover 1 week washout	Complex regional pain syndrome	16	Lidocaine IV infusions targeted to deliver plasma concentrations of 0.5, 1.0, 1.5, 2.0, and 3 mcg/ml	Diphenhydramine 70–80 mg	100-VAS	At 20 min	NA	NA
Wu et al. (51)	Crossover 1 day washout	Postamputation pain, stump pain	22	Lidocaine 1 mg/kg bolus and a 4 mg/kg iv infusion for 40 min	Diphenhydramine, 10 mg bolus iv + 40 mg infusion	100-VAS	Up to 30 min	36.5 (23.5)	50.1 (25.5)
Kvarnström et al. (39)	Crossover 1 week washout	Peripheral neuropathic pain (trauma, surgery, compression)	12	Lidocaine 1.0 mg/kg for 10 min and then 1.5 mg/kg for 30 min	0.9% saline	10-VAS	Up to 150 min	NA	NA
Attal et al. (43)	Crossover with 2 weeks washout	Trauma, PHN	22	Lidocaine 5 mg/kg x 30 min	0.9% saline	100-VAS	At 60 min	19 (22)	38 (22)
Kvarnström et al. (41)	Crossover 4 days washout	Traumatic spinal cord injury	10	Lidocaine 1.0 mg/kg for 10 min and then 1.5 mg/kg for 30 min	0.9% saline	10-VAS	Up to 150 min	NA	NA
Finnerup et al. (42)	Crossover 6 days washout	Trauma or disease of the spinal cord or cauda equina	12	Lidocaine 5 mg/kg x 30 min	0.9% saline	100-VAS	At 35 min	42.67 (28.86)	59.42 (18.6)
Gottrup et al. (46)	Crossover 2 days washout	Peripheral neuropathic pain	19	Lidocaine 5 mg/kg x 30 min	0.9% saline	100-VAS	Up to 40 min	45 (29)	49 (25)
Tremont-Lukats et al. (56)	Parallel	Peripheral neuropathic pain	31	Lidocaine at 1, 3, and 5 mg/kg/h as 6 h infusion	0.9% saline	100-VAS	Up to 10 h	NA	NA
Viola et al. (55)	Crossover 4 weeks washout	Painful diabetic neuropathy	15	Lignocaine 5 and 7.5 mg/kg over 4 h for 4 weeks	0.9% saline	MPQ	At day 14	NA	NA
Gormsen et al. (54)	Crossover 3–11 days washout	Peripheral nerve injury	13	Lidocaine 5 mg/kg ×30 min	0.9% saline + vitamin B	100-VAS	Up to 24 h	NA	NA
Vlainich et al. (53)	Parallel	Fibromyalgia	30 (15 each group)	Lidocaine 240 mg diluted in 125 mL infused over a period of 1 h, once a week, for 4 weeks	0.9% saline	10-VAS	At 4 weeks	4.1 (2.3)	4 (2.1)
Park et al. (19)	Crossover 2 weeks washout	Neuropathic pain of failed back surgery syndrome	18	Lidociane 1 mg/kg and 5 mg/kg at 60 ml/h	0.9% saline	100-VAS	Up to 60 min	NA	NA
Stavropoulou et al. (18)	Crossover 2 days washout	Trigeminal neuralgia	20 (*n* = 40)^*^	Lidocaine 5 mg/kg ×1 h in two sessions	0.9% saline	10-VAS	Till post infusion	1.46 (1.37)	3.33 (2.02)
Albertoni et al. (16)	Parallel	Fibromyalgia	38 (19 each group)	Lidocaine 240 mg diluted in 125 mL infused over a period of 1 h, once a week, for 4 weeks	0.9% saline	10-VAS	At 4 weeks	3.3 (1.6)	4.4 (2.7)
Kim et al. (17)	Parallel	PHN or Complex regional pain syndrome type II	42 (21 each group)	Lidocaine 3 mg/kg infused over a period of 1 h, once a week, for 4 weeks	0.9% saline	10-VAS	At 4 weeks	2.9 (2.53)	4.74 (2.67)
Liu et al. (52)	Parallel	PHN	183	Lidocaine 5 mg/kg ×1.5 h	0.9% saline	10-VAS	Up to 4 weeks	NA	NA

*SD, Standard Deviation; VAS, Visual analog scale; PHN, Post herpetic neuralgia; h, Hours; NA, Not available; wk, week; mins, minutes; MPQ, McGill Pain Questionnaire*.

* *Each participant underwent two sessions of treatment and placebo*.

**Figure 3:**
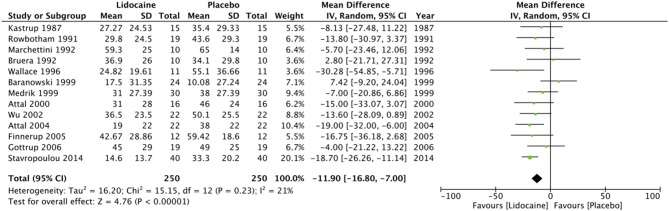
Forrest plot of IV lidocaine vs. placebo for pain in the immediate post-transfusion period.

**Figure 4:**
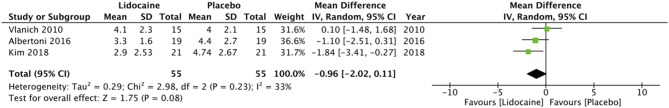
Forrest plot of IV lidocaine vs. placebo for pain persistent pain relief.

Pain scores from the immediate post-infusion period were available from 13 studies (11, 12, 14, 18, 42, 43, 45–51). Data on 250 patients from these studies was pooled to estimate the effect size. Our analysis indicates that lidocaine is superior to placebo in relieving neuropathic pain in the early post-infusion period (MD = −11.9; 95% CI: −16.8 to −7; *p* < 0.00001). Heterogeneity was non-significant (*I*^2^ = 21%, *p* = 0.23) (Figure 3). Multiple infusions of lidocaine over a period of 4 weeks, however, has no significant effect on relieving neuropathic pain (MD = −0.96; 95% CI: −2.02 to 0.11; *p* = 0.08) (Figure 4).

### Adverse Events

**Figure 5:**
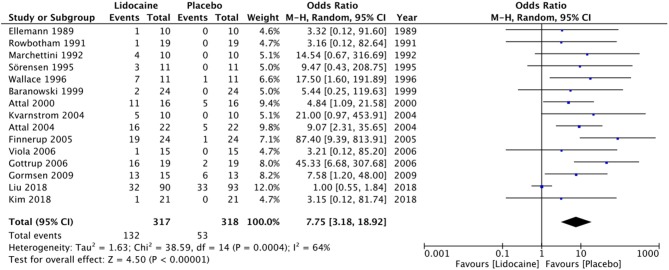
Forrest plot of adverse events with IV lidocaine vs. placebo.

Lightheadedness, somnolence, peri-oral paresthesia, nausea, headache, dysarthria, dry mouth, and metallic taste were some of the most common side effects noted by the trials (Table 2). Data was available from 15 studies (11, 13, 17, 42–46, 49, 50, 52, 54, 55) for a meta-analysis on adverse events from IV lidocaine usage. Three hundred seventeen patients received lidocaine, while 318 patients received a placebo. One hundred thirty-two patients (41.64%) in the lidocaine group experienced adverse events, compared to 53 patients (16.66%) in the placebo group. Our analysis shows that IV lidocaine is associated with a significant number of adverse events, compared to a placebo (OR = 7.75; 95% CI: 3.18–18.92; *p* < 0.00001) (Figure 5).

**Table 2:** Adverse events reported in included trials.

**References**	**Number of adverse events**		**Adverse events reported**
	**Lidocaine (*n*/*N*)**	**Placebo(*n*/*N*)**	
Kastrup et al. (14)	0	0	None
Ellemann et al. (13)	1/10	0/10	Transient drowsiness
Rowbotham et al. (11)	1/19	0/19	Nausea & Lightheadedness (1)
Bruera et al. (12)	0	0	None
Marchettini et al. (45)	4/10	0/10	Lightheadedness (4)
Sörensen et al. (44)	3/11	0/11	Nausea & perioral numbness (2); drowsiness, dysarthria, & tremor (1)
Wallace et al. (49)	7/11	1/11	Lightheadedness (6), Nausea (1)
Baranowski et al. (50)	2/24	0/24	Circumoral paresthesia (2)
Medrik et al. (47)	NA	NA	Dizziness, nausea, drowsiness, paresthesia, weakness, headache, palpitation
Attal et al. (48)	11/16	5/16	Lightheadedness/dizziness (7), somnolence (5), nausea/vomiting (3), dysarthria/garbled speech (3), malaise (2), headache (1), tinnitus (1), blurred vision (1), palpitation (1), facial numbness (1), dry mouth (1)
Wallace et al. (40)	NA	NA	Lightheadedness, Sedation, and dry mouth
Wu et al. (51)	0	0	None
Kvarnström et al. (39)	NA	NA	Somnolence, lightheadedness, out-of-body sensation changes in hearing/vision, nausea, itching, unpleasant experience, paresthesia
Attal et al. (43)	16/22	5/22	Lightheadedness, perioral numbness, and garbled speech
Kvarnström et al. (41)	5/10	0/10	Somnolence, perioral numbness (2)
Finnerup et al. (42)	19/24	1/24	Somnolence (11), dizziness (7), dysarthria (7), lightheadedness (7), blurred vision (3)
Gottrup et al. (46)	16/19	2/19	Tiredness (7), Nausea (4), Feeling drunk (3), Paresthesia (3), Blurred vision (3), Dizziness (2), Changed taste (3), Dysarthria (3), Headache (2), Dry mouth (2)
Tremont-Lukats et al. (56)	NA	NA	Lightheadedness (10), perioral numbness & headaches (6); nausea (4), diplopia (3), incoordination (3), throat tightness (3)
Viola et al. (55)	1/15	0/15	Lightheadedness (1)
Gormsen et al. (54)	13/15	6/13	Headache (2), Oral paresthesia (3), Dizziness (3), Somnolence (2), Memory impairment (3), Discomfort in the head (2), Fatigue (5), Feeling abnormal (2), Dry mouth (8), Nausea (3), Muscle spasms (2)
Vlainich et al. (53)	0	0	Not reported
Park et al. (19)	0	0	Not reported
Stavropoulou et al. (18)	NA	NA	Somnolence (13), Dry mouth (5), Dizziness (5), Headache (3), Feeling flushed (2), Confusion (1), Dysarthria (1), Tinnitus (1)
Albertoni et al. (16)	NA	NA	Nausea, vomiting, dizziness, drowsiness, paraesthesia, constipation, and dry mouth
Kim et al. (17)	1/21	0/21	Chest discomfort (1)
Liu et al. (52)	32/90	33/93	Drowsiness (5), Headache (6), Dizziness (19), Vomiting (2), Dry mouth (14), Metallic taste (2), Numbness (3)

*n/N, number of events/total number of participants; NA, not available; figures in parenthesis indicate number of patients reporting the side-effect*.

## Discussion

Chronic pain can be broadly classified into three categories of causation: (1). Due to tissue disease or damage (nociceptive pain), (2). due to somatosensory system disease or damage (neuropathic pain), or (3). a combination of nociceptive and neuropathic pain (mixed pain). Clinically, characteristics of neuropathic pain include the presence of burning/shooting/crawling/electric type of pain, abnormal sensation, or hypersensitivity (allodynia or hyperalgesia), and paraesthesia. Patients usually complain of spontaneous pain but some might also report evoked pain (57). These characteristics are not diagnostic for neuropathic pain, but indicate a strong possibility for it. While research indicates that there is damage of neuronal pathways in neuropathic pain, there are several mechanisms involved in its genesis. These mechanisms are independent of disease etiology, as the same mechanism can be seen in different diseases (57). In consideration of this theory, a large number of studies with different etiologies of pain were included in our review.

Regarding the actual mechanisms of pain initiation, research suggests that nerve injury results in an abnormal rate of proliferation and activation of sodium channels. Sodium channels produce uncontrolled persistent discharges resulting in a central hyperexcitable state. Such ectopic discharges can be initiated along the injured nerve, in the dorsal root ganglion, and in the peripheral neuromata (58, 59). The mechanism of action of IV lidocaine involves the alteration in the activation of sodium channels leading to a modification in pain response. Due to its sodium channel blocking action, lidocaine decreases peripheral nociceptor sensitization, and central hyperexcitability (60). The anti-inflammatory property of lidocaine also reduces circulating inflammatory cytokines, which are involved in the processes of secondary hyperalgesia and central sensitization. These actions of IV lidocaine occur at levels much lower than those required to produce a nerve conduction blockade (61).

According to our systematic review, a large number of clinical trials have tested IV lidocaine for neuropathic pain. However, the majority of these studies enrolled few patients (<30) and reported the use of a diverse range of lidocaine dosages and infusion times. Studies also assessed pain scored after various time periods. To consolidate data for the purposes of a quantitative analysis, we divided the studies into two groups. The first group consisted of studies reporting the efficacy of IV lidocaine in the immediate post-transfusion period while the second group consisted of studies where lidocaine was transfused over a period of 4 weeks to study its long-term, persistent effect. In the most recent systematic review of 2005 (15), data on 187 patents in the lidocaine group and 186 patients in the placebo group was analyzed and lidocaine was found to be superior to a placebo in the treatment of neuropathic pain (*p* = 0.003). From our systematic literature search, we found 11 new studies published after 2005 which were included in this review. Three (16, 17, 53) out of these 11 studies were classified into group two for the quantitative analysis of a long-term, persistent effect of IV lidocaine. Of the eight remaining studies, pain data was extractable as mean and SD from three of the studies. These were included in the meta-analysis of pain relief in the immediate post-transfusion period. Our results based on the data on 250 patients in each group shows that IV lidocaine is more effective than a placebo in relieving neuropathic pain in the immediate post-infusion period (*p* < 0.00001). From our analysis of data on 55 patients in lidocaine and placebo groups, we found that IV lidocaine does not have a long-term, persistent effect after repeated weekly infusions over a period of 4 weeks (*p* = 0.08). Although animal studies have reported that systemic lidocaine has long-term benefits for pain relief (62), our analysis suggests that the effect of lidocaine in humans is transient and does not last over a long period of time. This may be explained by the pharmacokinetics of the drug. The onset of action of lidocaine is between 30 and 60 min and the effects can last from 2 to 6 h after the end of the infusion, following which the analgesic effect rapidly wears off (17).

The correct dosage needed for pain relief with IV lidocaine is debatable. While some studies have recorded pain relief after 1 mg/kg (50) and 2 mg/kg (35) infusions, others have reported no significant pain relief from lower doses of lidocaine (56). Tremont-Lukats et al. (56), in a trial comparing 3 doses of IV lidocaine (1, 3, and 5 mg/kg), concluded that lidocaine infused at 1 and 3 mg/kg/h was no better than a placebo in relieving neuropathic pain. Another debatable subject is the optimal rate of lidocaine administration. The majority of studies (13, 14, 44, 48) have used a high infusion rate of 167 μg/kg/min (5 mg/kg over 30 min); however, an infusion rate >50 μg/kg/min may lead to adverse cardiovascular events (17). Conversely, other studies (56) that used a very low infusion rate of 14 μg/kg/min over 6 h found the treatment was effective. However, in a clinical outpatient setting, a 6-h infusion is not practical. The lack of clear guidelines on dosage and infusion rates can be attributed to the complex nature of neuropathic pain and the methodologic variability of the clinical trials conducted on IV lidocaine infusion. Considering the heterogeneity in past literature, further evidence in the form of well-controlled homogenous RCTs are required to provide clarity on dosage and infusion rates of IV lidocaine.

All studies included in our review used IV lidocaine in patients with normal conduction as demonstrated by electrocardiography (ECG) and normal serum electrolyte concentrations, as IV lidocaine can cause serious adverse events, such as cardiac arrhythmias and hemodynamic instability. The participants were monitored for changes in ECG and blood pressure throughout the infusion period. Side effects reported were mostly minor in nature, such as lightheadedness, somnolence, peri-oral paresthesia, nausea, headache, dysarthria, dry mouth, and metallic taste. Our analysis suggests that patients receiving IV lidocaine are more prone to adverse events compared to a placebo. However, it is notable that no serious adverse events were reported by any of the trials.

Some limitations of our review need to be mentioned. Firstly, not all studies included in the review were suitable for quantitative analysis. This was mainly due to a lack of clear presentation of the data by the trials. Secondly, there was a lack of standardization of lidocaine dosages and infusion rates across studies. Thirdly, not all studies included were high quality trials. Ten of the studies were rated as “medium quality” based on the quality assessment scale. Fourthly, it was not possible to conduct a subgroup analysis based on the specific etiology of neuropathic pain considering the limited number of studies available and the wide range of etiologies reported.

Nevertheless, our review is the first update conducted since the 2005 meta-analysis (15) on the use of IV lidocaine for neuropathic pain. Our study indicates that while IV lidocaine is effective in pain control in patients with neuropathic pain in the immediate post-transfusion period, it does not have a long-lasting, persistent effect. IV infusions of the drug are associated with an increased risk of side effects compared to a placebo. However, the risk of serious adverse events is negligible. Further, well-designed RCTs evaluating the effects of various dosages and infusion periods of IV lidocaine are required to provide clear guidelines on its clinical use.

## Data Availability

All datasets analyzed for this study are included in the manuscript/supplementary files.

## Author Contributions

BZ and KL conceived and designed the study. BZ, XZ, HW, and SW were involved in literature search and data collection. QZ analyzed the data. BZ wrote the paper. KL reviewed and edited the manuscript. All authors read and approved the final manuscript.

### Conflict of Interest Statement

The authors declare that the research was conducted in the absence of any commercial or financial relationships that could be construed as a potential conflict of interest.
